# The Duration of a Co-Occurring Sound Modulates Visual Detection Performance in Humans

**DOI:** 10.1371/journal.pone.0054789

**Published:** 2013-01-23

**Authors:** Benjamin de Haas, Roberto Cecere, Harriet Cullen, Jon Driver, Vincenzo Romei

**Affiliations:** 1 Wellcome Trust Centre for Neuroimaging at UCL, University College London, London, United Kingdom; 2 UCL Institute of Cognitive Neuroscience, University College London, London, United Kingdom; 3 Centro studi di ricerche in Neuroscienze Cognitive, Alma Master Studiorum, Universita’ di Bologna, Bologna, Italy; Radboud University Nijmegen, The Netherlands

## Abstract

**Background:**

The duration of sounds can affect the perceived duration of co-occurring visual stimuli. However, it is unclear whether this is limited to amodal processes of duration perception or affects other non-temporal qualities of visual perception.

**Methodology/Principal Findings:**

Here, we tested the hypothesis that visual sensitivity - rather than only the perceived duration of visual stimuli - can be affected by the duration of co-occurring sounds. We found that visual detection sensitivity (d’) for unimodal stimuli was higher for stimuli of longer duration. Crucially, in a cross-modal condition, we replicated previous unimodal findings, observing that visual sensitivity was shaped by the duration of co-occurring sounds. When short visual stimuli (∼24 ms) were accompanied by sounds of matching duration, visual sensitivity was decreased relative to the unimodal visual condition. However, when the same visual stimuli were accompanied by longer auditory stimuli (∼60–96 ms), visual sensitivity was increased relative to the performance for ∼24 ms auditory stimuli. Across participants, this sensitivity enhancement was observed within a critical time window of ∼60–96 ms. Moreover, the amplitude of this effect correlated with visual sensitivity enhancement found for longer lasting visual stimuli across participants.

**Conclusions/Significance:**

Our findings show that the duration of co-occurring sounds affects visual perception; it changes visual sensitivity in a similar way as altering the (actual) duration of the visual stimuli does.

## Introduction

Time is a fundamental dimension across sensory modalities. Manipulating the temporal characteristics of a stimulus in one modality can affect time perception in other modalities causing a discrepancy between physical stimulus timing and its perception (cf. [1]for a review). Changing the physical flutter rate of a clicking sound changes the apparent flicker rate of a flashing light (e.g. [2], [3]). More recently it has been shown that the perceived number of visual events in a rapid sequence can be biased towards the number of co-occurring sounds [4], that the timing of a static sound can determine the perceived direction of visual apparent motion [5] and that the perceived temporal closeness of visual events can be biased by temporally shifted auditory events [6].

More specifically, several studies have described cross-modal effects on subjective duration perception (e.g., [7]–[10]). The first finding in this regard was that perceived auditory stimulus durations are expanded relative to perceived visual durations, and that the perceived duration of audiovisual stimuli is more similar to the one for auditory stimuli [7]. This kind of auditory dominance is thought to reflect higher reliability of the auditory system for temporal judgments (e.g. [9]). In line with, and extending, this hypothesis is the observation that this kind of auditory dominance can be reversed for a high ratio of visual to auditory stimulus reliability ([3], c.f. [6]). The relative expansion of perceived auditory durations has been interpreted to reflect a faster auditory ‘pace maker’ mechanism for a presumed internal clock (c.f. e.g. [9]). However, this could not explain all findings regarding cross-modal effects on duration perception. For instance, the perceived duration of visual flashes was found to critically depend on the duration of co-occurring sounds [8], [10], [11]. If brief flashes are accompanied by sounds, they can be perceived as temporally shorter or longer than a unimodal flash of same duration, depending on the duration of the co-occurring sound [10]. This has been interpreted to reflect a ventriloquist-like capture of visual stimulus on- and offsets by sounds, which would translate to changes in the timing of ‘mode switch closures’ in the above mentioned model of an internal clock [10].

In a recent study [11] we replicated the effect of auditory stimulus duration on perceived visual duration. We asked participants to judge which of two brief flashes lasted longer. Stimulus durations were adjusted to a standard of 55 ms vs. the individual threshold for unimodal duration discrimination. We found that sensitivity was significantly enhanced when stimuli were accompanied by sounds of congruent durations. Audiovisually incongruent stimulus pairings led to significantly *de*creased performance (i.e. pairing the longer sound with the shorter flash and vice versa). These effects were abolished for asynchronous onsets of flashes and sounds and greatly reduced for sounds of much longer duration than the visual flashes. We interpreted this as evidence that the effect necessitates multisensory integration (rather than the participants simply ignoring the task demand to judge visual rather than auditory stimulus durations). We further speculated that if the duration of a sound affects the perceived duration of a concurrent visual stimulus, this might not be confined to duration ‘judgements’, but affect the actual duration of the visual perception itself, as if the visual representation was stretched in time by a longer lasting sound.

Accordingly, here we test the hypothesis that the duration of co-occurring sounds affects visual perception itself by impacting objective visual performance for non-temporal visual stimulus qualities. If slightly longer sounds result in sustained perception for co-occurring peri-threshold visual events, this should not only affect duration judgments for these kinds of stimuli but also improve visual sensitivity for these events, similar to what one would expect for physical lengthening of the visual stimulus duration.

In the present study our aim was to test whether pairing a visual stimulus with a longer lasting sound would yield prolonged perception of the visual stimulus. If so, sensitivity for the visual stimulus should be facilitated for longer sounds, similar to the visual detection improvement expected for a genuinely longer lasting visual stimulus.

Furthermore we anticipated any such effect to be restricted to a critical time window of audiovisual integration. Previous studies point to the importance of cross-modal stimulus onsets falling within a time window of about 100 ms for audiovisual binding to occur [12], [13]. As mentioned above, our recent findings point to a similar time window regarding the effect of prolonging sounds on visual stimulus duration judgments. If the duration of sounds is stretched too far beyond the visual stimulus offset, the effect disappears [11]. We therefore aimed to parametrically vary the duration of co-occurring sounds up to about 100 ms and add an additional data point for a sound duration presumed to fall well beyond this temporal window of integration. Our hypothesis was that if there was an effect of sound duration on visual sensitivity, sensitivity would continuously increase with sound duration but fall back to baseline level for the longest sound duration purposefully chosen to fall beyond the window of audiovisual integration.

## Materials and Methods

### Ethics Statement

All participants gave written informed consent to take part in this study, according to the Declaration of Helsinki. The study was approved by the UCL Research Ethics Committee, project ID no 1893/005.

### Participants

Twenty-eight participants were recruited for this experiment (mean age 25.1 years, range 25–30 years; 19 females, all right handed). All reported normal or corrected visual acuity and normal hearing. All participants were paid for their time.

### Apparatus

Stimuli were presented on a 21′CRT display (Sony GDM-F520) in a darkened room. Participants sat with their head in a chin rest at 65 cm viewing distance. Video resolution was 1600 × 1200, with a screen refresh rate of 85 Hz. Two small stereo PC speakers were placed on either side in front of the monitor. Stimulus control and data recording were implemented on a standard PC, running a MATLAB script using functions of Psychophysics Toolbox 3 [14]. Un-speeded manual two-choice responses (see below) were given using a standard PC numeric pad.

### Stimuli

In each trial, a rectangle containing dynamic white noise (mean luminance: 4.8 cd/m^2^; size: 23.5×17.7 degrees of visual angle) was presented for two consecutive intervals, each lasting 1059 ms (i.e. 90 frames at a video refresh rate of 85 Hz), with a SOA (stimulus onset asynchrony) of 300 ms between the two displays. A fixation dot extending 0.22 degrees in visual angle was superimposed at the middle of the noise rectangle, which was centred on the screen. The fixation dot was visible throughout the whole experiment, and changed its colour from red to green as a ‘go’ signal for responses in between trials. The target visual stimulus was a transparent Gabor patch (alpha blending factor of.6) which was briefly flashed at 353 ms after the onset of the first or second dynamic noise rectangle. The Gabor patch was composed of a 2D sinusoidal luminance grating with spatial frequency of 2.69 cycles per degree visual angle within a Gaussian amplitude envelope of standard deviation 10. It was embedded in the white noise visual stimulation with its centre position 1.4 degrees visual angle below the fixation dot. The luminance amplitude of the Gabor patch was set to individual threshold and its duration varied with experimental conditions (see below).

The auditory stimulus was a 400 Hz sinusoidal pure tone sampled at 44.1 kHz with 8 different durations (∼24, ∼36, ∼48, ∼60, ∼72, ∼84, ∼96 and ∼190 ms). Sound level was set to a ∼70 dB(A). In the audiovisual trials of the main experiment, sound stimuli of equal duration were presented at 353 ms after the onset of either of the white noise rectangles.

### Procedure: Visual Titration

Only visual stimuli were presented during this part of the experiment. In each trial a Gabor patch of ∼24 ms duration was embedded in one of two consecutive white noise displays with its midpoint at 1.4 degrees below fixation. In between trials the red fixation dot turned green, indicating participants should respond as to whether the Gabor patch was presented in the first or second white noise interval by pressing “1” or “2” on a keyboard. Across trials we pseudo-randomly varied the luminance amplitude of Gabor patches following a constant stimuli design (8 steps within a range of peak luminance measurements between 4.8 cd/m^2^ to 6.3 cd/m^2^). This allowed us to identify the luminance threshold for each participant individually.Participants completed 2 blocks, each containing 14 trials of each of the 8 luminance amplitudes tested, i.e. 112 trials in total.

The threshold was defined as the luminance amplitude allowing participants to correctly answer in 60% of the cases. In order to determine threshold luminance, we entered each individual visual titration curve into a sigmoid function and picked up the luminance value corresponding to the 60% accuracy on the sigmoid.

### Procedure: Main Experiment

**Figure 1:**
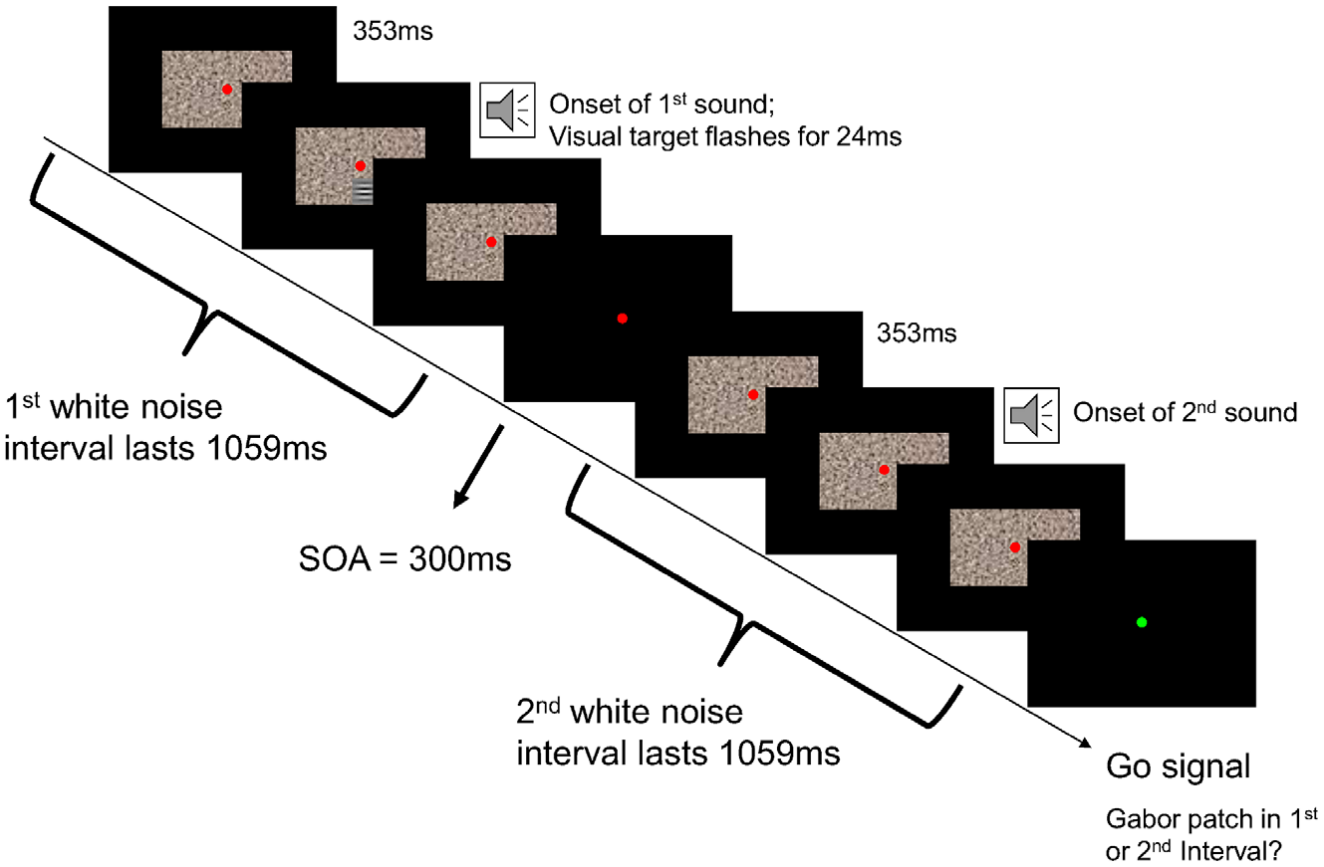
Illustration of a trial in the main experiment. Participants fixated a central red dot, while two consecutive intervals of dynamic white noise were presented on the screen. In either the first or second interval a Gabor patch was flashed for ∼24 ms at 1.4 degrees visual angle below fixation. The target flash appeared equiprobably in the first and second interval (each interval lasting 1059 ms, with the onset of the Gabor flash at 353 ms). In the example depicted the target flash appears in the first interval. Additionally, in both intervals a sound of variable duration (∼24 to ∼190 ms) was presented (sound onset 353 ms after interval onset for both intervals). In trials of a second, visual only, condition no sounds were played and the duration of Gabor patch flashes was variable (∼24 to ∼190 ms, matching sound durations in the audiovisual condition). After stimulus presentation the fixation dot turned green, indicating participants should report whether they perceived the Gabor patch in the first or second interval by button press.

In the main experiment, participants were again presented with a consecutive pair of dynamic white noise rectangles. Again, a Gabor patch was embedded in either the first or second white noise interval, and participants had to indicate whether they saw the flash in the first or second interval after each trial. The luminance amplitude of the target Gabor patch was set to a fixed value corresponding to the individual threshold, determined by the titration procedure for each participant. Trials in the main experiment fell in two conditions in pseudo-random order. In *visual* trials the flashing Gabor patch lasted for one of eight different durations (∼24, ∼36, ∼48, ∼60, ∼72, ∼84, ∼96, ∼190 ms), varying pseudo-randomly between trials. In *audiovisual* trials, the Gabor patch flash duration was fixed at ∼24 ms. While in visual trials no sound occurred, audiovisual trials additionally contained a pure tone auditory stimulus which was played twice with onsets at 353 ms after the first and second white noise interval respectively (i.e. synchronous with the Gabor patch onset in the target interval and at the matching time point during the non-target interval). The duration of tones pseudo-randomly varied between trials, corresponding to the same eight durations that the flashes could have in the unimodal visual condition (∼24, ∼36, ∼48, ∼60, ∼72, ∼84, ∼96 and ∼190 ms). Tone durations were always equal for the first and second interval of a given trial. Participants were instructed that auditory stimuli were irrelevant for the purpose of the task and therefore to ignore them. Participants completed 6 blocks of 80 trials each for a total of 30 stimuli per duration tested. The procedure is illustrated in Figure 1.

### Data Analysis

For each participant we computed visual sensitivity (d’) for the visual detection task, we did this independently for each of the visual and corresponding auditory-visual durations using standard formulae [15].

To address extreme cases (where false rates were zero) we adjusted all d’ values as suggested in [16]: false alarm rates were calculated as the number of false alarms +0.5, divided by the number of no-signal trials plus one (and, equally, hit rates as the number of hits +0.5, divided by the number of signal trials plus one; c.f. [17]). d’ was analysed using repeated-measure analysis of variance (ANOVA), with Condition (visual only and audiovisual) and Duration (∼24, ∼36, ∼48, ∼60, ∼72, ∼84, ∼96 and ∼190 ms)as within subjects factors followed up by paired t-tests where appropriate.

## Results

### Effects of Visual and Auditory Stimulus Duration on Visual Sensitivity

**Figure 2:**
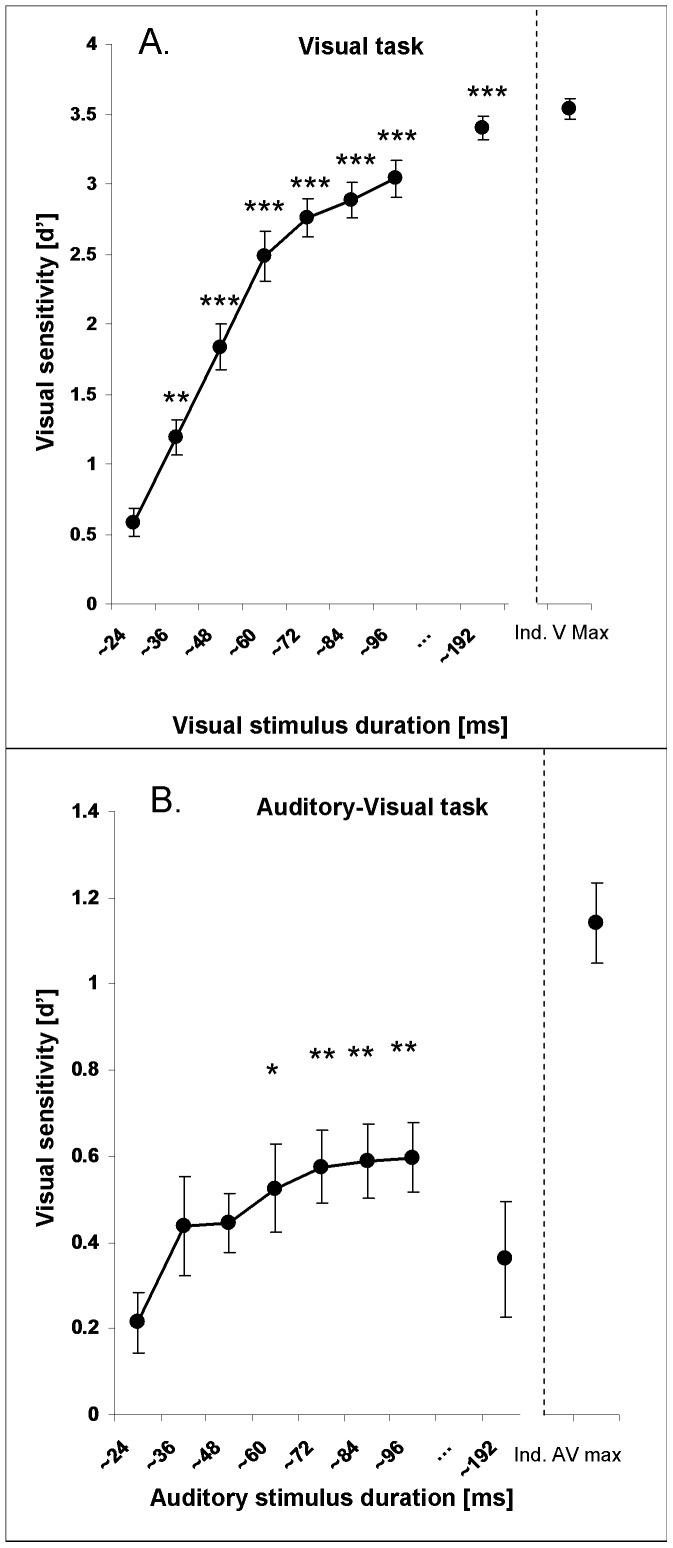
Effect of stimulus durations on visual sensitivity. Mean visual discrimination sensitivity (d’, SEM indicated) for varying visual stimulus durations (2A, upper panel) and for visual stimuli of fixed duration (∼24 ms), paired with auditory stimuli of varying durations (2B, lower panel). Asterisks indicate significant enhancement in visual sensitivity relative to the shortest (audio-) visual stimulus (leftmost data point in figures 2A and 2B) (* p<.05, ** p<.01 ***p<.001; all Bonferroni corrected). The rightmost data point represents the average maximum of the visual sensitivity enhancement across participants in the visual task (namely ‘Ind. V max’, Figure 2A) and audiovisual task (namely ‘Ind. AV max’, Figure 2B).

The sensitivity (d’), group means and standard errors are shown in figure 2A (visual stimuli alone) and 2B (audio-visual stimuli). Note the increase in sensitivity in Figure 2A as a function of visual stimulus length and the corresponding increase in sensitivity in Figure 2B for auditory stimuli of ∼60, ∼72, ∼84 and ∼96 ms, before falling back towards baseline level at ∼190 ms. Please refer to tables 1 and 2 for the complete set of signal detection results.

**Table 1:** Hit rates (HIT), false alarm rates (FA) and criteria (c) for the visual condition.

Duration	HIT % (S.E.M.)	FA % (S.E.M.)	c (S.E.M.)
**24** **ms**	70% (±3%)	48% (±3%)	−0,21 (±0,07)
**36** **ms**	76% (±3%)	34% (±3%)	−0,15 (±0,07)
**48** **ms**	84% (±3%)	23% (±3%)	−0,16 (±0,07)
**60** **ms**	88% (±2%)	11% (±2%)	0,009 (±0,06)
**72** **ms**	94% (±1%)	9% (±2%)	−0,06 (±0,05)
**84** **ms**	94% (±1%)	6% (±2%)	0,01 (±0,04)
**96** **ms**	96% (±1%)	6% (±2%)	−0,05 (±0,04)
**190** **ms**	99% (±1%)	3% (±1%)	−0,05 (±0,05)

Cells contain the mean and standard error of the mean (S.E.M.) across participants. Signal trials were defined as the ones in which the visual stimulus was displayed during the first interval.

**Table 2:** Hit rates (HIT), false alarm rates (FA) and criteria (c) for the audiovisual condition.

Duration	HIT % (S.E.M.)	FA % (S.E.M.)	c (S.E.M.)
**24** **ms**	64% (±2%)	53% (±3%)	−0,21 (±0,07)
**36** **ms**	65% (±4%)	50% (±3%)	−0,21 (±0,08)
**48** **ms**	62% (±3%)	42% (±3%)	−0,06 (±0,07)
**60** **ms**	65% (±3%)	44% (±3%)	−0,13 (±0,05)
**72** **ms**	64% (±4%)	41% (±3%)	−0,06 (±0,08)
**84** **ms**	68% (±3%)	45% (±3%)	−0,18 (±0,06)
**96** **ms**	65% (±3%)	40% (±3%)	−0,06 (±0,06)
**190** **ms**	62% (±4%)	49% (±3%)	−0,15 (±0,08)

Cells contain the mean and standard error of the mean (S.E.M.) across participants. Signal trials were defined as the ones in which the visual stimulus was displayed during the first interval.

The 2×8 ANOVA showed a main effect of Condition (F(1,27) = 563.73; p<0.000), a main effect of Duration (F(7,189) = 60.7; p<0.000) and a significant interaction between these two factors (F(7,189) = 46.92; p<0.000). We broke down our analysis by the factor Condition, thus testing the factor Duration for visual only trials and audiovisual trials separately. For visual only trials we found a significant effect of Duration (F(7,189) = 92.17; p<0.0000). Paired t-tests showed that compared to the shortest visual stimulus duration (∼24 ms, our baseline measure: BSL), all other visual stimulus durations enhanced visual sensitivity (all *p*s<0.004, Bonferroni corrected). Crucially, also the repeated-measure ANOVA for audiovisual trials showed a significant effect of auditory stimulus Duration on visual sensitivity (F(7,189) = 2.29; p = 0.029).

Paired t-tests showed that compared to the shortest, baseline audiovisual stimulus (∼24 ms), visual sensitivity was enhanced for auditory stimulus durations of ∼60 ms (t(27) = −3.08; p = 0.03, Bonferroni corrected), ∼72 ms (t(27) = −3.75; p = 0.006, Bonferroni corrected) ∼84 ms ((t(27) = −4.06; p = 0.005, Bonferroni corrected) and ∼96 ms (t(27) = −3.84; p = 0.005, Bonferroni corrected). All other auditory durations (∼36, ∼48 and ∼190 ms) did not significantly differ from our BSL (all ps>0.23, Bonferroni corrected).The maximum of the auditory-duration induced visual sensitivity enhancement was smaller (∼0.6 d’; 7% increase relative to audiovisual BSL) than the enhancement induced by a genuinely longer lasting visual stimulus (∼3 d’; 35% increase relative to visual enhancement). Furthermore, sensitivity for the shortest (∼24 ms) audiovisual stimulus was significantly lower than for the shortest (∼24 ms) unimodal visual stimulus (t(27) = −3.36, p<.05, Bonferroni corrected), while sensitivity for all other sound durations was not significantly different from sensitivity for the shortest unimodal visual stimulus (all p values >.24).

### Correlation between Effects of Visual and Auditory Stimulus Duration

The magnitude of visual sensitivity enhancement for prolonged sounds relative to the shortest sound (i.e. BSL corrected values) varied considerably across participants (range: 0 to 2.35 d’, mean.93 d’; SD:.51 d’). The same was true with regard to the magnitude of enhancement for genuinely prolonged visual stimuli relative to the shortest visual stimulus, i.e. BSL corrected values (range: 0.86 to 3.84 d’, mean: 2.95 d’; SD:.69 d’). There were individual differences with regard to both processes: visual sensitivity enhancement by prolonging visual stimulus duration and by prolonging the duration of co-occurring sounds (cf. [18]–[21] for individual differences in audiovisual integration). We reasoned that the size of these effects would be correlated across participants if they stem from similar mechanisms.

Furthermore, participants also differed with regard to the particular sound duration for which they showed maximum visual sensitivity enhancement. We speculated that this reflected genuine trait-like differences between participants, such as the individual width of the multisensory window of integration (cf. [20], [21]). Based on this assumption we reasoned that the maximum visual sensitivity enhancement (relative to BSL) for a given participant would be the best indicator for this participant’s effect size relative to other participants. We refer to this *individual* peak auditory enhancement as ‘the maximum audiovisual sensitivity enhancement’ (Ind. AV max).

In a similar way we calculated the *individual* ‘maximum visual sensitivity enhancement’ (Ind. V max) in the unimodal condition. Note that the *absolute* size of both ‘Ind. V max’ and ‘Ind. AV max’ are likely to be inflated and thus non-informative per se. Still they appear as the ‘fairest’ way of quantifying *relative* individual effect sizes without biasing towards a particular window of integration.

**Figure 3:**
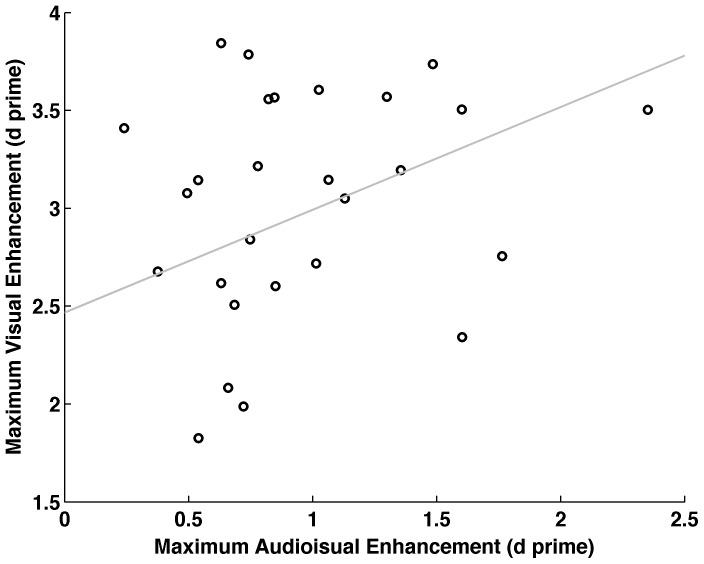
Correlation between visual and audio-visual enhancement. Correlation between individual peak auditory-induced enhancement (Ind. AV max) and peak visually-induced enhancement. Note that the maximum effective auditory and visual durations varied between participants and were thus determined on an individual basis (c.f. Methods for details).

We therefore tested the correlation between ‘Ind. AV max’ and ‘Ind. V max’. The individual maxima in duration induced enhancement were significantly correlated between both conditions (r = .38, p<.05; see Figure 3).

## Discussion

### Sound Duration and Visual Sensitivity

We found that visual sensitivity depended on the duration of co-occurring auditory stimuli. Specifically, visual detection sensitivity (d’) for a ∼24 ms visual flash was significantly enhanced for auditory stimuli whose durations were between ∼60 and ∼96 ms, as compared to performance at baseline (matching auditory duration of ∼24 ms). However, no such visual sensitivity enhancement was found for an auditory stimulus lasting much longer than this critical time window (∼190 ms). A rather surprising aspect of our results is that the baseline level of performance in the audiovisual condition was significantly lower than the unimodal baseline performance. Participants’ detection performance became significantly worse when a ∼24 ms flash was accompanied by an auditory stimulus of matching duration (as compared to no accompanying sound).

Our results are consistent with previous findings that auditory stimuli can bias duration judgments for co-occurring visual stimuli (e.g. [7]–[11]). Here, we show for the first time that the duration of auditory stimuli also impacts objective visual performance for non-temporal visual stimulus qualities.

We previously proposed that effects of auditory duration on visual duration judgements reflect cross-modal binding processes [11]. A visual event is perceived as longer when paired with a slightly longer lasting auditory event that is perceived as part of the same multisensory event [11]. If this reflects a genuine effect of sustaining visual perception, it should affect duration judgements as well as non-temporal qualities of visual perception, including detection sensitivity for visual stimuli.

Our current findings support this hypothesis and characterize the enhancement of visual sensitivity for sounds of longer duration within a restricted time window. This time window (∼60–96 ms) is consistent with previous findings regarding critical time windows for audiovisual integration (see e.g. [12], [13]).

### Lower Baseline Performance in the Audiovisual Condition

Despite the predicted pattern of results within the audiovisual condition, a comparison across conditions yielded a surprising result in need of explanation. Lower baseline performance in the audiovisual condition was neither predicted by our hypotheses, nor the results of previous studies. Generally, the mere presentation of auditory stimuli during a visual task can modulate visual performance (e.g. [4], [12], [22]–[29]) as well as responses in early visual areas [13], [30]–[35]. Even studies using very similar stimuli and paradigms found a detection sensitivity *enhancement* for audiovisual vs. visual stimuli, rather than a detrimental effect [26], [36].

In Noesselt et al. [26] participants had to decide in each trial, whether a Gabor patch was flashed in a cued peripheral region of interest or not. As in our experiment, flashes were quite brief (17 ms) and could be accompanied by a sound of matching duration. The presence or absence of sounds was not informative (sounds were as likely to be played in no-signal as in signal trials). Flash intensity was thresholded to 55–65% (low intensity) or 85–95% correct (high intensity) for unimodal visual stimuli. The presence of sounds yielded a significant detection sensitivity enhancement for low intensity stimuli. This condition was very similar to our experiment, with the only differences being that our stimuli were embedded in dynamic noise patterns and we used a two interval forced choice design, rather than a simple detection design. The latter difference could in theory be of importance – simple detection designs are more prone to biases in decision criterion and sound induced performance enhancements could thus be due to criterion shifts. Participants in this study [26] showed indeed a conservative bias for low intensity stimuli, but the improvement in the audiovisual condition was in objective performance (d’) and not accompanied by a shift towards a more liberal criterion. The differences in results between this study and ours are thus unlikely to derive from the differences in task design.

The design of Chen et al. [36] was even closer to ours. Here, like in our study, participants had to decide in which of two intervals a Gabor patch was flashed for 17 ms, and stimuli were embedded in dynamic noise. The only difference was that in this study stimulus frames were interleaved with frames of noise, while our stimuli were superimposed with the noise mask (see above, Methods: Stimuli). Chen et al. [36] measured stimulus intensity thresholds for fixed steps of noise intensities and compared the resulting threshold curves in the presence vs. absence of a non-informative, co-occurring sound with matching duration. The co-occurring sound led to significantly lowered detection thresholds, but this effect was restricted to one out of seven noise intensities. Further, an example set of psychometric functions provided (their Figure 2) points to the possibility that the sound-induced enhancement for stimulus intensities around threshold (i.e. 75% correct for unimodal stimuli) might be much less pronounced, or even reversed for lower stimulus intensities (yielding performance levels of 55–65% that we aimed for in our thresholding performance). Taken together, baseline performance enhancement for audiovisual stimuli – in the particular design we used – seems to be rather subtle and dependent on specific combinations of performance level, signal intensity and noise intensity.

Still, to our knowledge, we are the first ones to report a significant detrimental effect of co-occurring sounds. Further research is needed to investigate this effect. One might speculate that very short sound transients can have a detrimental effect on visual sensitivity due to modality specific latencies in neural processing and the tendency of the subjective point of audiovisual synchrony to be shifted towards a visual lead [37]. Depending on the nature of cross-modal interactions, a sound-induced boost in visual neural activity might precede the onset of visually evoked activity. This in turn could lower the signal to noise ratio of visual stimulus evoked activityHowever both of the studies discussed above ([26] and [36]) found sensitivity *enhancement* for even shorter audiovisual stimuli than ours. Further research could investigate this effect in a systematic way by parametrically varying perceptual performance levels, signal intensity and noise intensity. Crucially, future studies could also test for a potential role of modality specific processing latencies by introducing and parametrically varying a temporal offset between flashes and sounds.

### Do Longer Sounds Affect Visual Sensitivity?

The pattern of results we observed can be interpreted in two major ways. One interpretation, consistent with our initial hypothesis would suppose a process lowering overall visual sensitivity in the presence of co-occurring sounds, and a second, counter-acting process of visual sensitivity enhancement for longer lasting sounds. An alternative interpretation would suppose a process lowering visual sensitivity that is exclusive to a sound of short, matching duration and would suppose no effect on visual sensitivity whatsoever for longer sounds. Although the latter interpretation appears simpler, we think our data are more in line with the first interpretation.

There are two aspects of our data that are hard to reconcile with the view that only the shortest sound duration had an effect on visual sensitivity. The first is the shape of the curve for visual sensitivity vs. sound duration (Figure 2 B). Just as we predicted, visual sensitivity gradually increased for longer durations, but fell back to baseline level for a duration purposefully chosen to fall outside the temporal window of integration (e.g. [12], c.f. Introduction). This drop to baseline level is expected under the hypothesis of prolonged sounds *within* the temporal window of integration enhancing visual sensitivity. But it is unexpected and hard to explain under the assumption that only sounds of matching duration had an effect on visual sensitivity. It would be interesting for future experiments to test visual sensitivity between 96 and 190 ms (for which we have no data). It would be particularly interesting to see whether visual sensitivity rises above unimodal baseline level before it drops off again.

The second aspect of our data supporting an enhancement of visual sensitivity due to prolonged sounds is the observed correlation between conditions. Across participants the maximum difference between visual baseline level and performance for prolonged visual stimuli correlated with the maximum difference between audiovisual baseline and performance for prolonged sounds. This correlation would fit with the hypothesis that prolonged sounds yield a sustained visual neural activity or visual perception. We expect participants gaining more from physically prolonged visual stimulus durations to equally gain more from cross-modally induced sustain of visual representations. In contrast, there is no explanation for this correlation under the assumption that only the shortest sound duration had an effect on visual sensitivity. Taken together, we view the first of our proposed interpretations to be the more likely one for the pattern of data we observed. But if auditory stimulus duration influences visual sensitivity, how does it do so?

### Potential Mechanisms for Sound Duration Dependent Visual Sensitivity Enhancement

As noted above, the mere presentation of auditory stimuli during a visual task can modulate visual performance (e.g. [4], [12], [22]–[28]) as well as responses in early visual areas [13], [30]–[35]. In light of these findings it seems reasonable to interpret our results as representing sustained visual activation corresponding to the duration of co-occurring auditory stimuli. A recent study by Romei et al. [38] found that the presentation of a brief auditory stimulus can phase-align oscillatory activity in the alpha frequency band over occipito-parietal areas and consequently modulate perception. These findings suggest a role for alpha oscillations in determining cross-modal effects on visual cortex excitability that might apply to our results. A critical time window of ∼60–100 ms would indeed correspond to one full alpha cycle and is likely to represent the temporal window for binding crossmodal information. Furthermore, the observed inter-individual variability in optimal duration of auditory stimuli could correspond to individual differences in oscillatory alpha frequency. Future studies should ascertain whether and to what extent the effects of auditory stimulus duration on visual sensitivity and duration judgments are due to oscillatory phase reset.

An alternative (but possibly compatible) mechanism mediating the effects of longer sounds on visual sensitivity would be sound-induced attention or arousal. The accumulated stimulus energy of a longer sound will exceed the one of a shorter sound and maybe this kind of stronger signal is better suited to guide temporal attention towards the visual stimulus. Note, however that this kind of temporal uncertainty reduction has been psychophysically tested and refuted as an explanation for visual sensitivity enhancement induced by the mere presence of co-occurring sounds [36]. Nevertheless, it could play a role in the duration dependent effects described here. If temporal attention guidance is (at least part of) the mechanism behind our findings, it would show interesting features of cross-modal integration. Its effects would be restricted to a temporal window of integration and would take place *after* the visual stimulus offset (note that the physical offset of the visual stimulus always preceded any physical differences in sound durations). The latter point underscores that any such effect of attention would be hard to distinguish from a cross-modal sustain of visual representations. The distinction between an explanation involving temporal attention and cross-modal effects might be artificial after all. Specifically, effects of cross-modal phase reset and of attention might work hand in hand, as suggested by recent neurophysiological results [32].
